# Trends in reproductive health in Israel: implications for environmental health policy

**DOI:** 10.1186/2045-4015-1-34

**Published:** 2012-08-28

**Authors:** Tamar Berman, Hagai Levine, Ronni Gamzu, Itamar Grotto

**Affiliations:** 1 Public Health Services, Israel Ministry of Health, Jerusalem, Israel; 2 Braun School of Public Health and Community Medicine, Hebrew University – Hadassah, POB 12272, Jerusalem, 91120, Israel; 3 Ministry of Health, Jerusalem, Israel; 4 Faculty of Health Sciences, Ben-Gurion University of the Negev, Beer-Sheva, Israel

**Keywords:** Reproduction, Fertility, Trends, Epidemiology, Health policy, Surveillance, Exposure

## Abstract

Nearly two decades ago, researchers first reported that endocrine disrupting chemicals in the environment were affecting reproductive health in the general population. The purpose of this article is to examine the evidence of adverse reproductive health trends in Israel and to explore implications for environmental health policy in Israel. We reviewed studies and data in Israel regarding trends in reproductive health indices, specifically: breast and testis cancer, hypospadias, sperm quality, male factor infertility, and age at menarche. The data provide some evidence of adverse reproductive trends in the Israeli population: an increase in testicular cancer from 1990 to 2007, a decrease in age at menarche from 1986 to 2000, an increase in the prevalence of male factor infertility, and some evidence of decreasing sperm counts. However, we note that much of the evidence is limited.

The policy implications of reported adverse reproductive health trends possibly related to environmental exposure have been radically different in Europe and the United States. In Europe, such reports led the Parliament of the European Community to adopt a resolution on endocrine disruptors, which emphasizes the application of the Precautionary Principle. U.S. Environmental Protection Agency policy is focused on screening chemicals for endocrine disrupting properties and does not specifically refer to the Precautionary Principle. To date, there has been no formal governmental policy or strategy in Israel regarding endocrine disrupting chemicals. Environmental health policy on endocrine disruptors requires integrating evidence on human reproductive health trends, evidence on adverse reproductive outcomes in wildlife and experimental systems, and data from biomonitoring studies. Despite gaps in evidence and current data, we support a precautionary approach to regulating potential endocrine disrupting chemicals and reducing public exposures, especially in sensitive groups such as children and pregnant women.

## Introduction

Nearly two decades ago, researchers first reported that endocrine disrupting chemicals (EDCs) were affecting reproductive health, citing evidence from experimental animal studies, investigations of wildlife populations, reports on increased incidence of certain pathologies in men and women, and adverse health outcomes in girls whose mothers were treated with the estrogenic drug diethylstilbestrol (DES) [1]. The recognition that early embryonic, fetal, or neonatal exposures to EDCs could lead to permanent health effects in adulthood was central to the claim that these chemicals were affecting reproductive health.

The group of compounds identified as endocrine disruptors is highly heterogeneous and includes, for example, industrial solvents/lubricants and their byproducts (e.g., polychlorinated biphenyls, dioxins), plastics (e.g., bisphenol A (BPA)), plasticizers (e.g., phthalates), pesticides (e.g., dichlorodiphenyltrichloroethane), fungicides (e.g., vinclozolin), pharmaceutical agents (e.g., DES), and natural chemicals found in human and animal food (e.g., phytoestrogens, including genistein and coumestrol) [2].

While the evidence for adverse reproductive outcomes (infertility, congenital malformations, and hormone-mediated cancers) from exposure to EDCs is strong [2], it is unclear whether exposures to these chemicals have impacted reproductive health in the general population in Israel.

In 2002, Israel had the highest rate of *in vitro* fertilization (IVF) cycles out of 53 countries participating in the World Collaborative Report on Assisted Reproductive Technology [3]. Data from the Department of Health Information at the Ministry of Health show that the rate of IVF treatment cycles including ICSI (per 100,000 women age 15–49) has increased in Israel from 45.5 in 1990 to 154.5 in 2007. Clearly, it is difficult to ascertain how much of the increasing demand for IVF treatments in Israel is attributable to economic and social factors (including state funding of fertility treatments) and how much is attributable to infertility influenced by other factors, including environmental exposures.

The purpose of this integrative article is to establish whether there is evidence of adverse reproductive health trends in Israel and to explore implications for environmental health policy in Israel. The first part of the paper will characterize Israeli databases containing data relevant to reproductive health trends in the Israeli population and will summarize trends in breast and testis cancer, hypospadias, male factor infertility, sperm quality, and age at menarche. The second part of the paper will explore the environmental health policy implications of these trends: How have the European Commission and the U.S. Environmental Protection Agency (EPA) responded to indications of adverse reproductive health trends in the population? What is the appropriate health policy response in Israel to possible adverse reproductive health trends?

### Part 1: Reproductive health trends in Israel

We identified the following Israeli databases as containing data relevant to reproductive health trends in the Israeli population (Table 1). In this review, we present data from the international literature, Ministry of Health publications, conference reports, and unpublished results.

**Table 1:** Continuous systematic databases in Israel relevant to reproductive health trends

**Subject**	**Method**	**Years**
Israel National Perinatal Registry	Report of live births and birth defects from hospitals in Israel; estimated 70% of total birth defects	1995 onwards
Israel National Cancer Registry	Report of diagnosed cancer cases; estimated 94 % of solid tumors and 85% of nonsolid tumors	1960 onwards
*In vitro* Fertilization (IVF) treatment cycles	Mandatory reporting from all IVF units	1990 onwards
Age at menarche	Population-based ongoing survey (army recruits)	1986 onwards
Health problems among adolescents	Mandatory examination of army candidates	1967 onwards

#### Cancer

**Figure 1:**
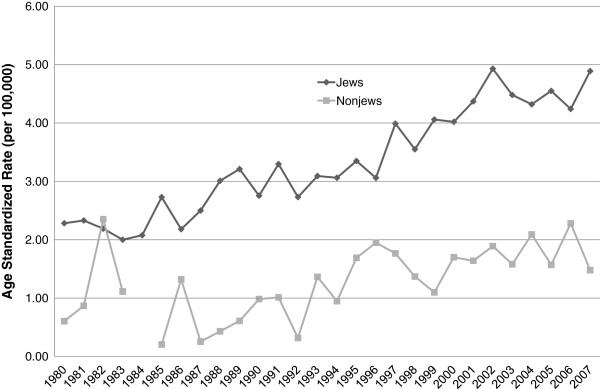
**Age Standardized Rate of Testicular Cancer in Israel, 1980–2007.** Reproduced from Bar-Chana [4].

Based on data from the Ministry of Health National Cancer Registry, which includes an estimated 94% of solid tumors and 85% of nonsolid tumors, age adjusted testicular cancer rates in Israel more than doubled in Jews and Arabs during 1990–2007, with age adjusted rates twice as high in Jews as in Arabs (Figure 1) [4]. Changes in incidence rates during this period were prominent in the Northern Negev, perhaps due to differences in environmental exposures [5].

A recent study of a cohort of military service candidates examined between 1967 and 2005, and linked to the Israel National Cancer Registry to obtain incident testicular cancer up to 2006, found lower risk for testicular cancer among men born in North Africa and Asia compared to European ancestry, but a steep increase in risk among next generations of migrants from North Africa. This study supports further research on the role of modern lifestyle and environment in the etiology of testicular cancer (Levine H et al., unpublished manuscript).

In situ breast cancer incidence has increased in both Jewish and Arab women from 1990 to 2007 (110% increase in Jewish women and 150% increase in Arab women) [6]. The increase since 1996 can be explained primarily by the increase in periodic mammography in the population (the National Mammography Program began in 1996). Invasive breast cancer incidence increased in Jewish women between the early 1990s and 1998 and then decreased steadily until 2007. Total breast cancer rates have been steady since 1998, as the decrease in invasive breast cancer rates in Jewish women has been matched by an increase in in situ breast cancer rates. In Arab women, invasive breast cancer rates more than tripled between 1990 and 2006, possibly due to improved diagnosis and socioeconomic changes in the population during that time (i.e., nutrition, number of births).

#### Hypospadias

Data from the Israel Birth Defects Registry, which includes reports on an estimated 70% of total birth defects in Israel, shows that hypospadias incidence rates in Israel were unstable between 1974 and 2008 [7]. Overall there was a very small increase during this period (31.73 per 100,000 in 1974–78 and 33.4 per 100,000 in 2004–2008). Rates of undescended testes in 2000–2008 have also been unstable [8]. It is unclear if the instability in reported hypospadias and undescended testes rates is due to data incompleteness or whether it reflects a real fluctuation in rates of these birth defects in the population.

#### Male factor infertility

In 1977, Dor and others reported on 655 infertile couples in Israel over a 15-year span and found that male infertility factors were involved in 28% of the cases [9]. Farhi and Ben-Haroush reported in 2011 that male factor infertility was the most common cause of infertility in 2515 couples referred to two infertility clinics in Israel between 1999 and 2007 [10]. The distributions of the causes of infertility were male factor infertility (45%), anovulation (37%), unexplained factors (20.7%), combined factors (18%), and tubal factors (18%), with each couple having one or more diagnosis. According to Farhi and Ben-Haroush, there has been a nearly twofold increase in the incidence of male factor infertility in similar infertile populations attending primary infertility centers evaluated 30 years apart in Israel [10]. Sella and others corroborated the finding that male factor infertility was the most common cause of infertility in a larger population and reported that male factor infertility was responsible for 48.80% of cases in 29,282 female members of Maccabi Health Services with definitive diagnoses of infertility between 1997 and 2010 [11].

#### Sperm quality

There have been few studies on trends in sperm quality in Israel. Almagor et al. showed a significant downward trend in sperm count and motility in Jerusalem men providing sperm samples for intrauterine insemination treatment during the years 1990–1999 [12]. Sperm counts declined by 5.2 ± 0.9 million sperm per year (*p* < .0001) and motility was reduced by 0.50 ± 0.14% per year (*p* < .0003). This trend remained strongly significant when men with normal parameters were evaluated separately, in order to control for the increase in oligoazoospermic patients in later years. In a longitudinal analysis, where each man’s earliest semen sample was compared to his latest one, a decrease in sperm counts was observed (decrease of 5.3 ± 4.5 million each year) although results were not significant.

Results of studies in sperm donors in Israel have been conflicting. One study, published in 1997, found an increase in total motile sperm count (7.74% per year, *p* < 0.0001) and a decline in normal morphology in semen from young Jerusalem donors between 1980 and 1995 (1.04% per year, p < 0.0001) [13]. The study was conducted in 188 men who donated sperm at the Hadassah Ein Kerem Hospital. Another study conducted at the Hadassah Mount Scopus Hospital compared 20 past donors (1995–1999) to 16 interim donors (2000–2003), and 22 recent donors (2004–2009) [14]. Over the study period, the average sperm parameters dropped from a concentration of 106 ± 25 million spermatozoa/ml with 79% ± 4.3% motility to 68 ± 14 million/ml with 66% ± 4.5% motile sperm (P < 0.0001, P < 0.0001, respectively). The total motile sperm count per ejaculate also decreased, from 66.4 ± 18.2 million to 48.7 ± 12 million (P < 0.005).

#### Age at menarche

**Figure 2:**
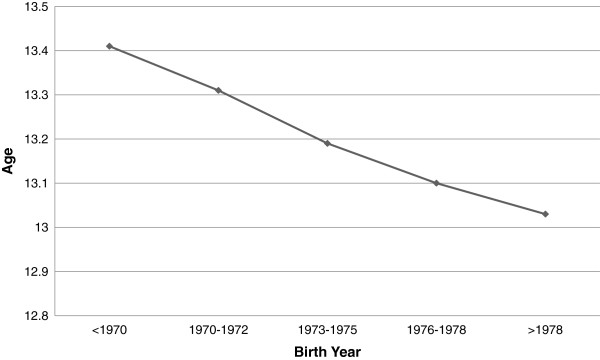
**Age at menarche in female recruits to the Israeli army, by birth year.** Reproduced from Chodick et al. [15].

A population-based survey among female recruits to the Israeli army from 1986 to 2000 found a decrease in age at menarche, unadjusted for BMI [15]. Reported mean age at menarche among the 11,392 recruits showed a monotonic trend of decrease over time, from 13.4 years for women born before 1970 to 13.0 years for those born after 1978 (Figure 2). Women born after 1978 were twice as likely as those born before 1970 to have had an early menarche (age 11 or earlier). Also, regression modeling showed a negative association between the recruit’s year of birth and their age at menarche, indicating a downward temporal trend of 4.2 months per decade in age at menarche. The trend was found for women of western and eastern origins and for all categories of paternal education. These results likely reflect the Jewish young adult population in Israel, as military service is compulsory, with the exception of Arabs and ultra-Orthodox Jews who are largely exempted from military service.

#### Summary of reproductive health trends

The data presented in the first part of this paper provide some evidence of adverse reproductive trends in the Israeli population: an increase in testicular cancer from 1990 to 2007, an increase in the prevalence of male factor infertility, a significant decrease in age at menarche in female recruits to the Israeli army from 1986 to 2000, and limited evidence of decreasing sperm counts.

However, we note that much of the evidence is limited. While the increase in testicular cancer in 1990–2007 is salient, additional types of cancer not associated with endocrine disruptors have increased in Israel during this time period. Data on changes in sperm quality in Israeli sperm donors is valuable, but such studies are inherently limited by the small sample sizes and the fact that sperm donors are not representative of the general population. The studies conducted by Dor and Farhi and Ben-Haroush on male factor infertility focused on specific clinics, which might introduce a certain selection bias; therefore it is unclear if the upward trend in male factor infertility reflects a national trend. In addition, it is possible that technological advances in diagnosis affected the difference in incidence of male factor infertility reported in 1977 and in 2011. Data on age at menarche in female recruits were unadjusted for BMI, and it is unclear if changes in obesity and life style during this period or other factors explain this trend.

In addition, other factors that are potentially related to reproductive health trends in Israel have changed in the population over the period that these adverse reproductive health trends were observed: increased recreational drug use [16]; a steep rise in obesity and overweight in youth [17]; major demographic shifts resulting from immigration trends [18]; and widespread soy consumption [personal communication], including soy based formula [19]. Increased recreational drug use, for example, may contribute to the increased prevalence in male factor infertility [20], while trends in prevalence of overweight may contribute to trends in age at menarche [21].

Despite the limitations in the data, the fact that adverse reproductive health trends have been reported in other populations worldwide suggests that there is some plausibility to the hypothesis that these trends are indeed occurring in Israel. Testicular cancer has been increasing worldwide in the majority of industrialized countries in North America, Europe, and Oceania, particularly among Caucasians [22]. In several European countries, including France, testicular cancer incidence has doubled since 1970 [23]. In addition, studies from European and U.S. populations indicate a trend toward younger ages of menarche and breast development in girls [24,25]. Finally, meta-analyses have indicated a decline in mean sperm concentrations since the 1930s [26,27] although these analyses have been challenged [28]. In Denmark, sperm counts in healthy young men are so low that researchers have predicted lower fertility rates in the future [29].

### Part II: Environmental health policy implications

Endocrine Disrupting Chemicals: EU and U.S. Policy Initiatives and Responses: The policy implications of reported adverse reproductive health trends possibly related to environmental exposure have been radically different in Europe and the U.S.

In 1996 scientists and policy-makers in Europe convened the Weybridge Workshop and reached the conclusion that “sufficient evidence exists for increasing testicular cancer rates, and the apparent decline in sperm counts in some areas was unlikely to be attributable to the known confounding variables.” Despite the fact that they concluded that existing exposure information was generally insufficient to definitely associate the human changes seen with chemical exposure, they noted that measures to reduce exposure to endocrine disrupters should be in line with the Precautionary Principle [30].

The EU Community Strategy for Endocrine Disrupters, which was adopted in 1998, emphasizes the application of the Precautionary Principle and calls on the Commission to identify substances for immediate action [31]. Actions that have been carried out by the Commission in relation to EDCs have included: a) development of a priority list of potential endocrine disrupting substances; b) monitoring studies, including a European wide biomonitoring study on phthalates in urine; c) research; and d) legislative actions such as restrictions on approval of EDCs for plant protection, legislation on food contact materials, including banning the use of BPA in infant feeding bottles, and new legislation on phthalates in toys [32].

In the U.S., the Endocrine Disruptor Screening Program Policy Statement from 1998 cited “conflicting reports … concerning declines in the quality and quantity of sperm production in humans over the last 4 decades, and … reported increases in certain cancers (e.g., breast, prostate, testicular)” but added that there is “considerable scientific uncertainty … regarding the actual causes of such effects.” [33] The U.S. Congress passed the Food Quality Protection Act and the Safe Drinking Water Act Amendments in 1996, requiring that the EPA screen chemicals and pesticides for their potential to produce effects similar to those produced by estrogen in humans. The EPA later expanded the screening program to include androgens and the thyroid system, and to include effects on fish and wildlife.

According to these acts, if a substance is found “to have an endocrine effect on humans, the Administrator shall … take action under such statutory authority as is available to the Administrator … as is necessary to ensure the protection of public health.” In other words, the statutes requiring testing for endocrine disruption provide no new process by which to regulate those chemicals. In addition, this approach demands scientifically *proving* harm in humans prior to regulating a chemical, without addressing the uncertainty involved in endocrine disruption and without mention of the Precautionary Principle [34].

Additional policy actions in the U.S. related to potential endocrine disruptors have included banning the use of phthalates in toys [35] and “taking reasonable steps to reduce human exposure to BPA in the food supply” [36].

Implications for Environmental Health Policy in Israel: In both the EU and the U.S., evidence regarding adverse reproductive health trends in the general population was considered when developing environmental health policy on EDCs. However, this data was considered within the broader context of evidence on the impact of EDCs on wildlife and experimental systems. To date, there has been no formal governmental policy or strategy in Israel regarding EDCs. Current actions in Israel have focused on three main aspects: monitoring, research/surveillance, and policy/legislation.

#### Monitoring

In the U.S., there is on-going surveillance of population exposure to environmental chemicals, including estrogen disrupting chemicals within the framework of the National Health and Nutrition Health Survey [37]. This surveillance supplies valuable data to assess the effectiveness of public health efforts to reduce exposure to environmental chemicals, to determine whether exposure levels are higher among susceptible subgroups, and to direct priorities for research on human health effects from exposure to these chemicals. The EU has recently launched a harmonized biomonitoring study on exposure to methyl mercury, cadmium, and phthalates, as well as environmental tobacco.

In Israel, the Ministry of Health recently conducted the first national biomonitoring study to measure exposures to BPA, phthalates, organophosphates, polyaromatic hydrocarbons, the phytoestrogens genistein and daidzein, and cotinine in the Israeli adult population [38]. The study involved collection of urine samples from 250 adults in Israel, with detailed demographic and dietary data, including a 24-hour recall and food frequency questionnaire. Data on urinary concentrations of environmental contaminants in the study participants indicate widespread exposure to environmental chemicals, including to synthetic and natural endocrine disrupting compounds. Further study will include analyses of predictors of exposure (intake of food items/use of consumer products), data that will aid in assessing possible sources of exposure and can be used to develop policy actions to reduce population exposure to these environmental chemicals. The Ministry of Health is also currently conducting a study on levels of persistent organic pollutants, including dioxins, in pooled breast milk samples.

#### Research/surveillance

Currently, there is lack of surveillance on reproductive health outcomes for the Israeli population. We emphasize the need for further study in Israel on reproductive health trends and on possible associations between adverse reproductive outcomes and exposures to environmental chemicals. Researchers at the Hebrew University of Jerusalem have recently initiated studies on associations between organophosphate exposure and endocrine outcomes related to male infertility, and on associations between prenatal exposure to phthalates and BPA and birth outcomes. We note that endocrine disrupting chemicals may interfere with endocrine systems other than the reproductive system and emphasize the need for study on trends of thyroid conditions, obesity, and diabetes and possible associations with exposure to environmental chemicals. We also note that many epidemiological studies on the associations between exposures to environmental chemicals and adverse reproductive outcomes have been hampered by the lack of individual exposure assessment.

#### Policy/legislation

The precautionary approach has been applied in Israel in a number of recent policy decisions concerning EDCs, including for example the ban on use of BPA in baby bottles and the decision to severely restrict the use of the herbicide atrazine. In the case of BPA, quantitative risk assessments showed that little or negligible risk was expected following normal use of baby bottles [39,40]. However, since there was considerable uncertainty regarding the effects of low dose chronic exposure, evidence of increased vulnerability in infants and children, and available alternatives, the Ministry of Health adopted a precautionary approach [41].

In addition, the Ministry of Health recently recommended the ban of the herbicides atrazine and simazine in Israel, based on concerns regarding possible effects on reproduction and fetal development from chronic low dose exposure via drinking water. In this case, current levels of these herbicides in drinking water in Israel are far below the Israeli Drinking Water Standard, suggesting that there is negligible risk to the population. In fact, the World Health Organization recently recommended an increase in the drinking water standard in light of evidence that atrazine is not carcinogenic in humans. However, given emerging suggestive evidence of adverse effects on reproduction and development at very low levels of exposure, and given that there were available alternatives, the Ministry of Health adopted a precautionary approach.

We argue that environmental health policy on EDCs should be informed by a broad range of evidence, including data on trends in reproductive health, evidence from wildlife and experimental systems, and biomonitoring data.

Despite gaps in evidence and current data regarding adverse effects of environmental chemicals on reproductive health in Israel, we support a precautionary approach to regulating EDCs and reducing public exposures, especially in sensitive groups such as children and pregnant women. We argue that this principle should be invoked following a) identification of potentially negative serious or irreversible effects resulting from a phenomenon, product, or procedure; b) a scientific evaluation of the risk; and c) evaluation of the extent of the scientific uncertainty [42]. We emphasize that the Precautionary Principle should be invoked in cases where scientific uncertainty or insufficiency of the data, or their inconclusive or imprecise nature, makes it impossible to determine with sufficient certainty the risk in question. As Wier et al. have argued, the appropriateness of applying the precautionary principle *increases* when the exposure or harm is widespread, when the incidence of the harm (i.e., observed health effect) is increasing and is otherwise unexplained; when the suspected harm is serious; when the suspected harm is not easily treatable or reversible; when the economic and social costs of removing the exposure are small relative to the suspected harm; when the health costs of removing the exposure are minimal; and when, in addition to the uncertain harms, there are known health, economic, or social harms caused by the exposure [43].

We note also that the public-health strategy for dealing with potential effects of EDCs must reflect governing national and international legalities. In the international trade environment in which Israel is obligated to operate by its treaty obligations (i.e., General Agreement on Tariffs and Trade – Uruguay Round-Non-Tariff Barriers, Sanitary and Phytosanitary Measures) there are restrictions on the ability to apply any measure without scientific justification [44].

In the cases of BPA in baby bottles and atrazine in drinking water, in-depth scientific evaluation of the potential risk revealed significant uncertainties that made it impossible to determine that current exposure levels would result in negligible or no risk to the general population. In addition, in both of these cases potential exposure of the general population was widespread and the potential health outcomes were both serious and irreversible. However, we note the significant challenges in application and logical consistency of the Precautionary Principle in environmental health policy. For example, with regard to food contact uses of BPA, the Ministry of Health, in light of European and U.S. policy, has yet to determine whether there is a case for applying the Precautionary Principle and restricting or banning this use.

## Conclusions

There is limited evidence of adverse reproductive trends in the Israeli population. However, relative absence of evidence regarding adverse effects of environmental exposure on Israeli reproductive health does not equal the evidence of absence of such a problem. This is especially true in Israel, where there has been little research on associations between environmental exposures in the general population and adverse reproductive outcomes. We emphasize the importance of applying the Precautionary Principle in environmental health policy, monitoring exposure of the population to environmental chemicals, and monitoring trends in reproductive health outcomes in Israel. The Ministry of Health is currently evaluating research and policy development needs.

## Competing interests

The authors have no competing interests to declare.

## Authors’ contributions

TB and HL conducted the data and literature search and drafted the manuscript. IG and RG made significant contributions to the text and provided insight regarding policy implications. All authors read and approved the final manuscript.

## Authors’ information

Tamar Berman, PhD, is currently the Chief Toxicologist for Environmental Health at the Ministry of Health.

Hagai Levine, MD, MPH, is a public health physician and epidemiologist at the School of Public Health, Hebrew University–Hadassah, and is a member of the Center of Excellence for Environmental Research on Agriculture and Health at the Hebrew University of Jerusalem.

Ronni Gamzu, MD, is a gynecologist who specializes in Health Management and has a PhD from Tel-Aviv University. He is currently the Director General of the Israel Ministry of Health. He is also an Associate Professor at Tel-Aviv University.

Itamar Grotto, MD, PhD, is a public health physician who also has a PhD from Ben-Gurion University of the Negev. He is currently the Director of the Public Health Services in the Israel Ministry of Health. He is also an Associate Professor at Ben-Gurion University of the Negev.
